# Screening of potential oxidative stress-related biomarkers and therapeutic drugs in rheumatoid arthritis based on integrative bioinformatics, machine learning, and molecular dynamics simulations

**DOI:** 10.3389/fmolb.2026.1804435

**Published:** 2026-03-23

**Authors:** Zhigang Shi, Wenzhuo Qi, Juyin Xue, Shuxu Zhang, Zhou Mu, He Wang, Bingrui Zhu, Peng Kong, Yingguang Han

**Affiliations:** 1 The First Clinical Medical School, Shandong University of Traditional Chinese Medicine, Jinan, Shandong, China; 2 Department of Minimally Invasive Orthopedics, The Affiliated Hospital of Shandong University of Traditional Chinese Medicine, Jinan, Shandong, China

**Keywords:** biomarkers, machine learning, molecular dynamics simulation, oxidative stress, rheumatoid arthritis

## Abstract

**Background:**

Rheumatoid arthritis (RA) is a prevalent autoimmune condition. Increasing evidence reveals that oxidative stress exerts an important effect in the pathogenesis of RA. This research aimed to systematically screen oxidative stress-related biomarkers for RA and further examine promising therapeutic drugs for RA.

**Methods:**

This research first obtained transcriptome data of RA from the GEO database and identified differentially expressed oxidative stress-related genes (DEOSGs). Subsequently, core biomarkers were identified by integrating weighted gene co-expression network analysis with three machine learning algorithms. Their diagnostic performance was assessed utilizing receiver operating characteristic curves, and a clinical predictive nomogram was established. Functional enrichment analysis was implemented to systematically elucidate the biological processes involving DEOSGs in RA, and immune infiltration analysis was conducted concurrently. Furthermore, a potential therapeutic small-molecule compound was screened leveraging the CMap database and validated through molecular docking and molecular dynamics simulation. Finally, the expression levels of the core genes were quantified and analyzed utilizing quantitative real-time polymerase chain reaction and Western blot in a primary human RA synovial fibroblast model.

**Results:**

Totally, 281 DEOSGs were identified. These genes were significantly enriched in pathways including the MAPK, AMPK, TNF, and Toll-like receptor signaling pathways. Based on the machine learning algorithms, three core genes were ultimately determined. The diagnostic model established on the basis of these genes demonstrated good diagnostic efficacy. Immune infiltration analysis revealed significant differences in the distribution of immune cell subsets between RA patient samples and normal control samples. Molecular docking and molecular dynamics simulation indicated that narciclasine exhibited good binding affinity with the target protein, and the stability of the binding complex was acceptable. Furthermore, experimental results from the *in vitro* cell model confirmed that the expression patterns of the core genes were consistent with findings from the bioinformatics analysis.

**Conclusion:**

This research preliminarily suggests that CXCL10, EDNRB, and MMP13 may serve as potential oxidative stress-related biomarkers for RA. Simultaneously, it predicts that narciclasine may be a promising candidate drug for RA treatment. These findings offer new insights into the pathogenesis, targeted intervention, and treatment development for RA.

## 1 Introduction

Rheumatoid arthritis (RA) is a systemic autoimmune condition featuring synovial inflammation (Zhang et al., 2023). Clinically, it typically presents with joint pain, swelling, and morning stiffness (Huang et al., 2025; Riitano et al., 2025). As the condition progresses, it can lead to progressive destruction of articular cartilage and bone tissue, ultimately resulting in joint deformity and functional loss (Pascual-García et al., 2025). The global prevalence of RA is approximately 0.5%–1%, and women face a substantially greater risk than men (Tang et al., 2025). RA can affect individuals of all ages, with a peak incidence around 60 years old (Zhang et al., 2025). The pathogenesis of RA is complex and is closely related to genetic predisposition, environmental triggers, and dysregulated immune networks (Okada et al., 2019; Ziembicki et al., 2025; Açıkalın Göktürk and Sanlier, 2025). Challenges remain although various treatment options have emerged, including disease-modifying antirheumatic drugs, biologics, and targeted drugs (Zhang et al., 2025; Jahid et al., 2023). Some patients experience poor treatment response, drug resistance, or long-term side effects (Liang et al., 2025; Szekanecz et al., 2024). Furthermore, the disease exhibits strong heterogeneity, lacks a curative treatment, and requires long-term standardized management (Watanabe et al., 2022; Aletaha, 2020). This not only imposes a heavy physical and psychological burden on patients but also creates substantial economic pressure on families and medical systems. Therefore, RA remains a pressing public health issue demanding in-depth research and prioritized prevention and control.

Oxidative stress exerts a vital driving effect in the pathological process of RA (Djordjevic et al., 2023; Quiñonez-Flores et al., 2016; Kaur et al., 2021). In individuals with RA, the levels of oxidative stress byproducts, including reactive oxygen species (ROS) and reactive nitrogen species, are substantially elevated in synovial tissue, synovial fluid, and peripheral blood (da Fonseca et al., 2019; Kundu et al., 2012; Veselinovic et al., 2014; Khojah et al., 2016). Concurrently, the function of the endogenous antioxidant system, encompassing superoxide dismutase and glutathione peroxidase, is markedly impaired (Mateen et al., 2016). This imbalance disrupts the body’s oxidant-antioxidant homeostasis, which directly damages lipids, proteins, and nucleic acids in synovial cells, chondrocytes, and osteocytes, inducing oxidative damage, apoptosis, or abnormal activation, thereby accelerating synovial hyperplasia and pannus formation (Mateen et al., 2016; Datta et al., 2014; Phull et al., 2018). Furthermore, excessive oxidative stress byproducts act as “inflammatory amplifiers.” They promote the secretion of proinflammatory factors including tumor necrosis factor (TNF)-alpha and interleukin (IL)-6 by initiating signaling pathways including NF-κB and MAPK, and simultaneously suppress the expression of anti-inflammatory factors including IL-10 and transforming growth factor beta (TGF-β) (Phull et al., 2018; Dröge, 2002; Sies et al., 2017). This imbalance further exacerbates abnormal infiltration and activation of immune cells, thus forming a vicious cycle of “oxidative stress and inflammatory response.” (Guo et al., 2024; Wang et al., 2022). Moreover, oxidative stress drives the initiation and perpetuation of autoimmune responses in RA by modifying autoantigens and disrupting immune tolerance (Kurien and Scofield, 2008; Oparaugo et al., 2023; Hatzioannou et al., 2021). It also aggravates progressive destruction of articular cartilage and bone tissue by inducing overexpression of matrix metalloproteinases (MMPs), thereby enhancing their degradation capacity for joint cartilage collagen (Cabral-Pacheco et al., 2020). Numerous clinical studies have confirmed that interventions targeting the regulation of oxidative stress can, to some extent, alleviate inflammatory responses and delay joint damage (Kondo et al., 2023; Chandimali et al., 2025). This finding further underscores the important value of oxidative stress as a core component of the pathomechanism of RA and a potential therapeutic target.

Recently, the rapid development of gene microarray and transcriptomics technologies has provided powerful technical support for deciphering the complex molecular regulatory mechanisms underlying various diseases (Lan et al., 2018). The current research adopted an integrative bioinformatics analysis strategy to screen transcriptome data for synovial tissues of individuals with RA and healthy controls from public gene expression databases, thus systematically identifying differentially expressed genes (DEGs). Subsequently, potential biomarkers were determined by focusing on oxidative stress-related genes (OSGs) through weighted gene co-expression network analysis (WGCNA) and machine learning. Finally, small-molecule drugs with therapeutic potential for RA were forecasted by leveraging the connectivity map (CMap) database, and their binding activity and stability were validated utilizing molecular docking and dynamics simulations. On the whole, these data not only enhance our knowledge of the pathogenesis of RA but also uncover critical molecular targets from the perspective of oxidative stress. Moreover, they offer an essential theoretical foundation and clues for the future establishment of novel treatment strategies against oxidative stress and related drug research.

## 2 Methods and materials

### 2.1 Data extraction and handling

In the current research, four human RA synovial tissue datasets (GSE55235, GSE55457, GSE77298, and GSE12021) were sourced from the Gene Expression Omnibus (GEO) database (https://www.ncbi.nlm.nih.gov/geo/). Details are summarized in Table 1. During the analysis, GSE55235, GSE55457, and GSE77298 were designated as the training set (66 samples in total), while GSE12021 served as the validation set (21 samples). The training datasets were merged utilizing R. Subsequently, the ComBat method was applied to eliminate batch effects and normalize the data. The resulting data were utilized for subsequent model construction. Additionally, OSGs with an association score >7 were identified from the GeneCards database (https://www.genecards.org). Ultimately, a total of 2,133 genes were obtained.

**TABLE 1:** Details of gene expression dataset.

Dataset	Platform	Tissue	Rheumatoid arthritis	Control	Experiment type	Class
GSE55235	GPL96	Synovium	10	10	Microarray	Training set
GSE55457	GPL96	Synovium	13	10	Microarray	Training set
GSE77298	GPL570	Synovium	16	7	Microarray	Training set
GSE12021	GPL96	Synovium	12	9	Microarray	Validation set

### 2.2 Identification of DEGs

The “limma” tool was employed for linear model fitting and constructing the comparison matrix for RA and healthy control samples. DEGs were identified leveraging the cut-offs of |log2FC| ≥ 0.585 and false discovery rate (FDR) < 0.05. The selection results were visualized with a volcano plot and a heatmap. Subsequently, DEGs and OSGs were intersected, ultimately yielding the differentially expressed oxidative stress-related genes (DEOSGs).

### 2.3 Functional enrichment analysis

Functional enrichment analysis was implemented utilizing the clusterProfiler tool in R to systematically illustrate the biological functions and potential regulatory pathways of DEOSGs. The analysis included Gene Ontology (GO) (covering the three domains: molecular function (MF), biological process (BP), and cellular component (CC)) and Kyoto Encyclopedia of Genes and Genomes (KEGG) enrichment. Markedly enriched terms were determined with a cut-off of FDR <0.05.

### 2.4 WGCNA

A gene co-expression network was built utilizing WGCNA. First, sample clustering was performed to identify and remove outliers. The pickSoftThreshold function was subsequently utilized to identify the optimum soft thresholding power within a range of 1–22 according to a scale-free topology fitting index (*R* ^2^) ≥ 0.9. With this soft thresholding power, an adjacency matrix was estimated and then converted into a topological overlap matrix (TOM). A distance matrix (1-TOM) was generated for hierarchical clustering of genes. Gene modules were initially selected leveraging the dynamic tree cut method with the smallest module (cluster) size of 200. Highly similar modules, characterized by module eigengene (ME) distances <0.3, were then merged. A module-trait association heatmap was generated by calculating the Pearson association coefficients and their corresponding P-values between MEs and the traits of interest. Finally, the critical module with the greatest association was intersected with DEOSGs, yielding a candidate gene set.

### 2.5 Determination of core genes based on machine learning methods

The current research integrated three classical machine learning algorithms for screening genes, namely least absolute shrinkage and selection operator (LASSO), support vector machine-recursive feature elimination (SVM-RFE), and random forest (RF) to accurately determine potential biomarkers for RA. A fixed random seed was uniformly set for all computational processes to ensure the stability of the analysis process and the reproducibility of the results. The LASSO was implemented utilizing the “glmnet” tool. The optimum regularization parameter λ was ascertained through a 10-fold cross-check strategy, and genes with nonzero coefficients corresponding to the smallest λ value were determined as preliminary candidate features. SVM-RFE analysis was implemented with the caret tool, employing a radial basis function as the kernel function. The optimum number of variables was ascertained by the root mean square error (RMSE) from the 10-fold cross-check, thus sequentially eliminating features with low contribution. A classification model was built based on the RF algorithm. Initially, a model containing 1,000 decision trees was constructed, and the curve of error versus the number of trees was plotted. The number of trees corresponding to the lowest out-of-bag error was identified and utilized to retrain the model. Subsequently, high-importance features were filtered utilizing a cut-off of MeanDecreaseGini >1.5. Finally, the intersection of genes determined by the three algorithms was taken as the consensus feature set, and a three-set Venn diagram was generated to visually present the overlapping results.

### 2.6 Establishment of the nomogram and analysis of receiver operating characteristic (ROC) curves

First, univariate analyses were implemented to generate ROC curves, and the area under the curve (AUC) was computed to ascertain the diagnostic effect of individual genes for the disease. Subsequently, a logistic regression model was established with the “rms” tool, and a nomogram was developed on the basis of this model. A total of 1,000 bootstrap resamples were conducted and AUC and 95% confidence intervals (95% CI) of the calibration curve and the model were calculated to validate the model performance. Meanwhile, decision curve analysis (DCA) was implemented with the rmda tool to quantify net clinical benefits of the model among various threshold probabilities. Additionally, boxplots were generated via the “ggplot2” tool to display the differential expression levels of the core genes in training and validation datasets.

### 2.7 Gene set enrichment analysis (GSEA)

GSEA was implemented for each critical hub gene to investigate potential pathways and mechanisms associated with RA. With the median expression level of the gene across samples as the threshold, genes were stratified into high-expression and low-expression groups. Single-gene GSEA was then executed utilizing the “clusterProfiler” tool to explore potential KEGG pathways.

### 2.8 Immune infiltration analysis

Immune infiltration analysis was implemented utilizing the CIBERSORT deconvolution algorithm. Based on the LM22 feature matrix, the relative ratios of 22 immune cell subsets within every individual sample were computed, and the robustness of the results was enhanced by 100 permutation tests. Variations in immune cell ratios between the RA and control groups were contrasted leveraging the Wilcoxon test. Moreover, Spearman’s rank association analysis was implemented to explore the correlations of the expression levels of critical genes with immune cell infiltration levels.

### 2.9 Prediction of potential small-molecule drugs

The CMap database (https://clue.io) provides a reliable tool for exploring interactions among drugs, genes, and diseases through association analysis of gene expression profiles. In the current research, the DEOSGs were uploaded to the database. Association scores (ranging from −100 to 100) were calculated by contrasting the input gene expression patterns with drug-induced ones in the database. The magnitude of these scores was closely related to the therapeutic potential of the drugs. Specifically, a lower score was associated with a stronger negative relation of the drug with the disease-related gene expression profile. This relation indicated a greater ability to reverse abnormal gene expression patterns in the disease and a higher likelihood of becoming a candidate therapeutic drug. Ultimately, 10 small-molecule drugs with the highest negative enrichment scores were determined for deep analysis.

### 2.10 Molecular docking

The molecular structures of small molecules were acquired from the PubChem database (https://pubchem.ncbi.nlm.nih.gov/), and the 3D crystal structures of the target proteins were acquired from the Protein Data Bank (http://www.rcsb.org/). The water molecules and original ligands were eliminated from proteins with PyMOL, and the locations and dimensions of the binding pockets were determined with the GetBox Plugin. The handled proteins and small molecules were loaded into AutoDock Tools 1.5.6 separately and converted to the PDBQT format. Molecular docking calculations were then implemented leveraging AutoDock Vina 1.1.2. Finally, the results of docking were presented and analyzed with PyMOL 2.6.0. A binding free energy of ≤ −5.0 kcal/mol indicated good binding activity between the small molecule and the target.

### 2.11 Molecular dynamics simulation

Molecular dynamics simulations were implemented in the current research utilizing GROMACS 2022. The GAFF2 parameters for the ligand were generated utilizing Antechamber and electric charges were assigned via the RESP method. Protein parameterization was executed leveraging the AMBER14SB force field. The system was embedded in a cubic box with a 1 nm edge distance utilizing the TIP3P water model. Na^+^ and Cl^−^ ions were leveraged for system neutralization. Long-range electrostatic interactions were treated utilizing the particle mesh Ewald (PME) method with a cutoff distance of 1 nm. Relevant force field parameters and PME settings were tuned in adherence to protocols of GROMACS. Bond constraints were applied utilizing the linear constraint solver algorithm. In advance of the molecular dynamics simulation, the system was subject to energy minimization involving 3,000 steps of steepest descent and 2,000 steps of conjugate gradient algorithms. The simulation was performed under the NPT ensemble for a total duration of 100 ns with an integration time step set to 2 fs. The temperature was kept at 310 K with the Nosé–Hoover thermostat, and the pressure was kept constant at 1 bar with the Parrinello–Rahman barostat. Upon completion of the simulation, key properties were computed, including the root mean square deviation (RMSD), root mean square fluctuation (RMSF), number of hydrogen bonds (HBonds), radius of gyration (Rg), and solvent accessible surface area (SASA).

### 2.12 Cell culture

The normal human primary synovial fibroblast cell line CP-H248 and the human primary rheumatoid arthritis synovial fibroblast cell line CP-H241 were purchased from Wuhan Yipu Biotechnology Co., Ltd. These cells were maintained in DMEM/F-12 medium (11320033, Gibco) supplemented with 10% fetal bovine serum (abs972, Absin) and 1% penicillin-streptomycin solution (U31-301C, YOBIBIO), and cultured in a humidified constant-temperature incubator at 37 °C with 5% CO_2_. Synovial fibroblasts at passage 3 were used for subsequent experiments.

### 2.13 Quantitative real-time polymerase chain reaction (qRT-PCR)

Total RNA was isolated from cells utilizing TRIpure Total RNA Extraction Reagent (EP013, ELK Biotechnology). Afterwards, 1 μg of purified RNA was quantified. First-strand complementary DNA (cDNA) was generated by reverse transcription utilizing EntiLink™ first Strand cDNA Synthesis Kit (EQ003, ELK Biotechnology). Amplification reactions were then implemented on a qRT-PCR system (QuantStudio 6 Flex, Life Technologies) with EnTurbo™ SYBR Green PCR SuperMix (EQ001, ELK Biotechnology). GAPDH was utilized as the reference gene, and the relative expressions of target genes were computed utilizing the 2^(–ΔΔCt)^ method. Primer sequences are provided in Table 2.

**TABLE 2:** Primer information.

Gene	Accession number	Base sequence (5′-3′)		Product length
GAPDH	NM_002046	Sense	TCGGAGTCAACGGATTTGGT	181 bp
		Antisense	TTCCCGTTCTCAGCCTTGAC	
CXCL10	NM_001565.4	Sense	GACCAATGATGGTCACCAAATC	149 bp
		Antisense	GCAGGGTCAGAACATCCACTAA	
EDNRB	NM_000115.5	Sense	GGGGTTCCAAAATGGACAGC	136 bp
		Antisense	GAAGCAAGCAGATTCGCAGA	
MMP13	NM_002427.4	Sense	GCACTTCCCACAGTGCCTAT	82 bp
		Antisense	AGTTCTTCCCTTGATGGCCG	

### 2.14 Western blot

First, total protein was isolated from cells using RIPA lysis buffer (CBW0011, COBIO) containing protease inhibitors. The lysate was processed by centrifugation at 12,000 × g for 10 min at 4 °C, and the supernatant was acquired. Protein concentration was ascertained with a BCA protein assay kit (CBW0020, COBIO). Subsequently, a 5× protein loading buffer (CBW0029, COBIO) was added at a volume ratio of 4:1, and the samples were denatured at 95 °C for 15 min. A total of 50 μg protein per sample was loaded with a volume of 5 μL, separated by SDS-PAGE, and transferred onto methanol-activated PVDF membranes (IPVH00010, Millipore) at a constant current of 300 mA for 40 min. The membranes were blocked with 5% BSA (V900933, MERCK) on a decolorizing shaker at room temperature for 1 h, and then incubated with diluted primary antibodies overnight at 4 °C: anti-CXCL10 (10937-1-AP, Proteintech, diluted 1:1000), anti-EDNRB (20964-1-AP, Proteintech, diluted 1:1000), anti-MMP13 (YA3143, MCE, diluted 1:1000), and anti-GAPDH (60004-1-Ig, Proteintech, diluted 1:10,000). On the subsequent day, the membranes were washed three times with TBST buffer (CBW0089, COBIO) for 5 min each at room temperature, and afterwards, exposed to HRP-conjugated secondary antibodies (diluted 1:5000) for 30 min at room temperature. Upon the completion of incubation, the membrane was rinsed again with TBST (CBW0089, COBIO). Finally, protein bands were visualized using an enhanced chemiluminescence kit (P0018M, Beyotime) and imaged with a ChemiScope 6100 imaging system (CLiNX, China). The grayscale values of target bands were quantitatively evaluated with AlphaEaseFC and GAPDH was utilized as the reference protein for normalization.

### 2.15 Statistical analysis

Bioinformatics-related analyses and visualization analyses were implemented in R 4.5.1. The organization, analysis, and graphical presentation of data obtained from *in vitro* experiments were implemented with GraphPad Prism (10.0). All experimental data were presented as the mean ± standard deviation. Either the Wilcoxon rank-sum test or the unpaired t-test was selected for comparisons of group differences based on the distribution features of the data. Correlations between variables were analyzed utilizing Pearson association analysis or Spearman’s rank association test. Additionally, the Benjamini–Hochberg correction method was applied to control FDR. P < 0.05 was considered statistically significant.

## 3 Results

### 3.1 DEOSGs selection

**FIGURE 1:**
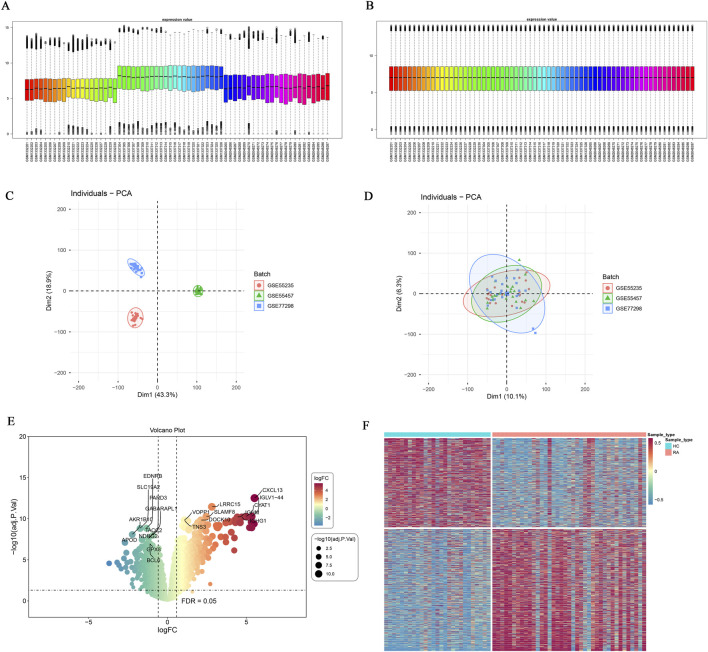
Data normalization and differential expression gene analysis. **(A)** Box plot of GEO dataset distribution before batch processing; **(B)** Box plot of GEO dataset distribution after batch processing; **(C)** PCA plot before batch processing; **(D)** PCA plot after batch processing; **(E)** Volcano plot of DEGs; **(F)** Heatmap of DEGs.

Prior to data analysis, batch effects in the gene expression data were eliminated. The distribution characteristics of data before and after correction were visually validated utilizing boxplots and principal component analysis (Figures 1A–D). On the basis of differential expression analysis of the database result, 1,605 DEGs were determined, involving 927 upregulated and 678 downregulated genes. Supplementary Table S1 lists the top 10 most significantly upregulated and top 10 most significantly downregulated DEGs. The variations in gene expression between the RA and control groups were clearly and intuitively presented leveraging volcano plots and heatmaps (Figures 1E,F). Moreover, 281 DEOSGs were ultimately obtained by intersecting DEGs with an OSG set. The complete list of DEOSGs is provided in Supplementary Table S2.

### 3.2 Functional enrichment analysis of DEOSGs

**FIGURE 2:**
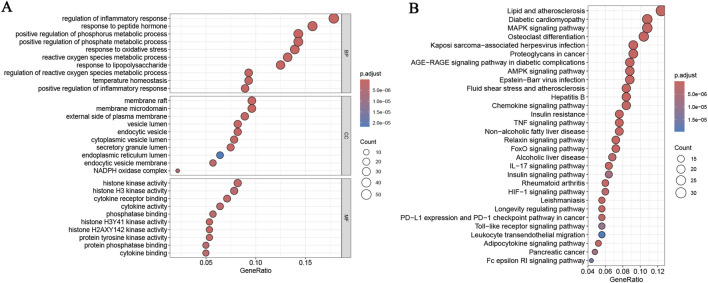
Functional enrichment analysis. **(A)** GO functional enrichment analysis of DEOSGs; **(B)** KEGG pathway enrichment analysis of DEOSGs.

A systematic functional enrichment analysis was implemented to illustrate the underlying mechanism of DEOSGs in the onset and progress of RA. GO enrichment results indicated that DEOSGs were markedly clustered in key functional terms, like regulation of inflammatory response, response to oxidative stress, regulation of reactive oxygen species metabolic process, endoplasmic reticulum lumen, NADPH oxidase complex histone kinase activity, and cytokine activity (Figure 2A). Furthermore, KEGG pathway analysis validated the pathway enrichment characteristics of these genes, demonstrating their substantial enrichment in pathways encompassing MAPK, AMPK, TNF, Chemokine, IL-17, and Toll-like receptor signaling pathways (Figure 2B). This finding offered critical direction for subsequent elucidation of the regulatory network of DEOSGs.

### 3.3 WGCNA analysis and determination of RA-related modules

**FIGURE 3:**
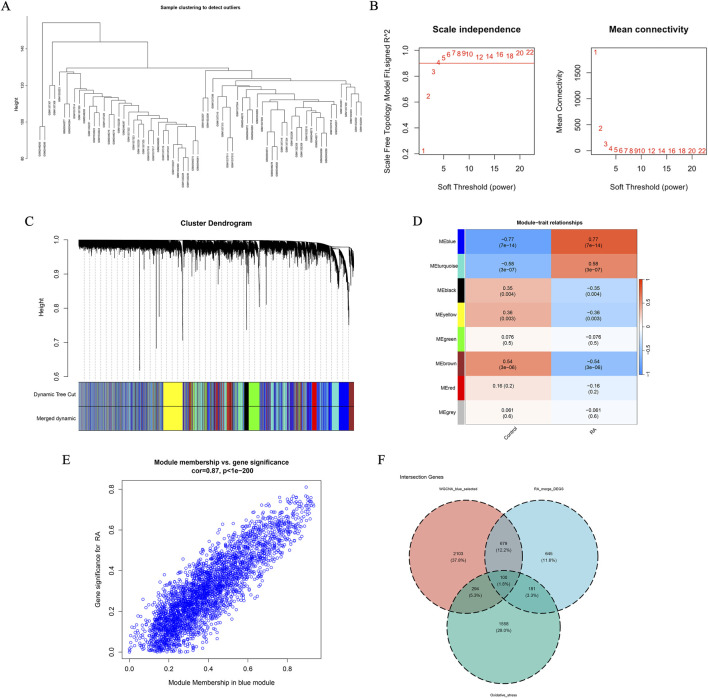
Construction of WGCNA. **(A)** Dendrogram of sample clusters; **(B)** Soft threshold power (left) and average connectivity (right) of WGCNA; **(C)** Gene dendrogram with multiple partitioning modules; different colors represent different modules (clusters); **(D)** Heatmap of correlation between module eigengene (ME) and clinical phenotype; **(E)** Scatter plot of correlation between module membership (MM) and gene significance (GS) of blue module; **(F)** Venn diagram of candidate genes.

WGCNA was implemented to further screen for feature genes of RA. Initially, cluster analysis of the included samples was executed, and the results indicated no outliers (Figure 3A). Subsequently, a scale-free co-expression network was established utilizing a soft thresholding power of 6 (Figure 3B). Eight distinct co-expression modules were determined via hierarchical clustering along with the dynamic tree cut algorithm (Figure 3C). Among them, the MEblue module exhibited the highest association with RA (r = 0.77, p = 7e−14) and contained a total of 3,176 genes (Figure 3D). A scatter plot of module membership and gene significance further validated the strong association between this module and the clinical phenotype of RA (Figure 3E). Finally, 100 candidate feature genes for RA were identified by intersecting the genes in the MEblue module with DEOSGs (Figure 3F).

### 3.4 Identification of core genes via machine learning

**FIGURE 4:**
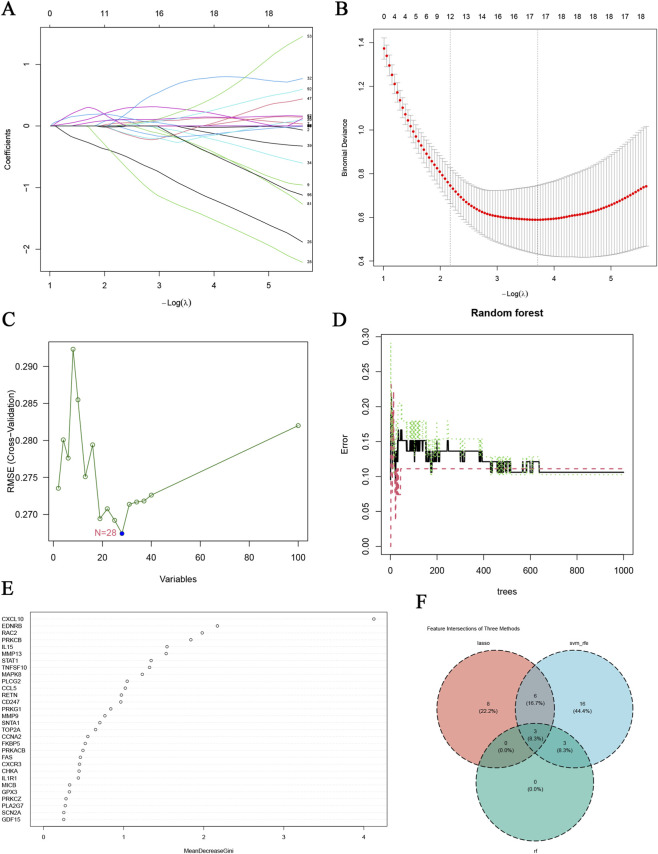
Machine Learning for screening core genes. **(A)** LASSO regularization path; **(B)** LASSO regression cross-validation curve; **(C)** Line chart of RMSE varying with the number of retained genes, with blue dots indicating the optimal number of genes corresponding to the minimum RMSE; **(D)** The curve of out-of-bag (OOB) error rate varying with the number of decision trees; **(E)** Mean decrease Gini histogram of the top 30 most important genes; **(F)** Venn diagram of the intersection of genes screened by three machine learning algorithms.

The current research adopted multiple machine learning algorithms to identify hub characteristic markers for RA. The LASSO regression algorithm was applied to screen candidate genes, yielding 17 genes (Figures 4A,B). The SVM-RFE algorithm was then used for gene selection, identifying 28 potential biomarkers (Figure 4C). Furthermore, the RF algorithm was implemented, and 6 core associated genes were identified at a selection threshold of MeanDecreaseGini >1.5 (Figures 4D,E). Intersection analysis was performed on the gene sets obtained from these three algorithms. Ultimately, three hub RA-related genes, including CXCL10, EDNRB, and MMP13, were identified (Figure 4F).

### 3.5 Establishment of a diagnostic prediction model

**FIGURE 5:**
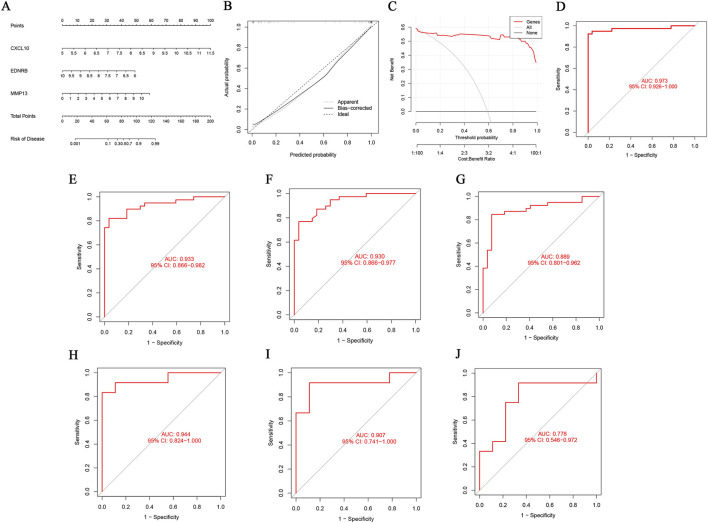
Construction and evaluation of the diagnostic model. **(A)** Construction of a nomogram model for the three core genes; **(B)** Calibration curve of the nomogram; **(C)** DCA of the nomogram; **(D)** ROC curve of the nomogram model; **(E–G)** ROC curves of CXCL10, EDNRB and MMP13 within the training set; **(H–J)** ROC curves of CXCL10, EDNRB and MMP13 within the validation set.

**FIGURE 6:**
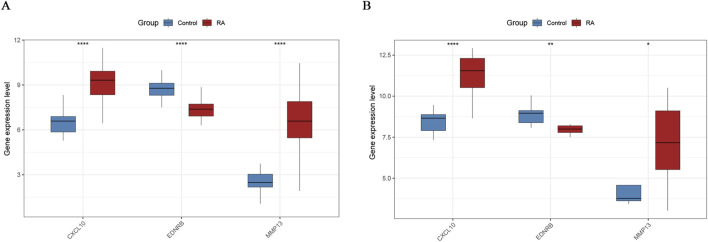
Expression patterns of core genes. **(A)** Expression patterns of CXCL10, EDNRB and MMP13 in RA and Control groups within the training set; **(B)** Expression patterns of CXCL10, EDNRB and MMP13 in RA and Control groups within the validation set. * denotes p < 0.05, ** denotes p < 0.01, **** denotes p < 0.0001.

A multivariate logistic regression was implemented to establish a diagnostic prediction model for RA in order to evaluate the diagnostic effect of the three core DEOSGs. Based on this model, a nomogram was generated to visually quantify the linear contribution of each gene’s expression level to RA risk (Figure 5A). The calibration curve, analyzed with 1,000 resamples, revealed minimal deviation between the observed and predicted risks of events (Figure 5B). Internal validation of the model yielded an AUC of 0.973 (95% CI: 0.926–1.000), suggesting high predictive accuracy (Figure 5D). Furthermore, DCA showed that this nomogram model could offer substantial clinical benefits for individuals with RA, confirming the practical utility of the model in clinical settings (Figure 5C). ROC curve analysis was implemented in the training set to ascertain the diagnostic utility of individual genes. The results revealed that AUCs for CXCL10, EDNRB, and MMP13 were 0.933, 0.930, and 0.899, respectively (Figures 5E–G). Independent validation was implemented in the external validation set GSE12021 to ensure the robustness of the findings. The validation results showed AUCs of 0.944, 0.907, and 0.778 for CXCL10, EDNRB, and MMP13, respectively (Figures 5H–J). Additionally, the expression patterns of the genes in RA and normal control samples were systematically analyzed. The results demonstrated that, both in the training and validation sets, CXCL10 and MMP13 were significantly upregulated in RA samples compared with controls, while EDNRB was significantly downregulated in RA samples (Figure 6).

### 3.6 Single gene GSEA of core genes

**FIGURE 7:**
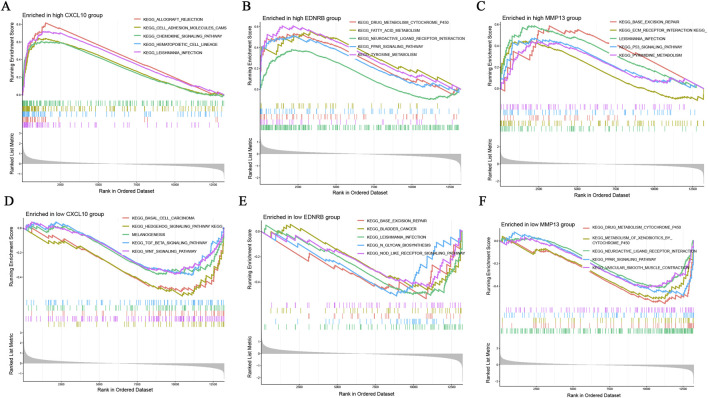
Single Gene GSEA of Hub Genes. **(A)** GSEA analysis of the CXCL10 high-expression group; **(B)** GSEA analysis of the EDNRB high-expression group; **(C)** GSEA analysis of the MMP13 high-expression group; **(D)** GSEA analysis of the CXCL10 low-expression group; **(E)** GSEA analysis of the EDNRB low-expression group; **(F)** GSEA analysis of the MMP13 low-expression group.

The single-gene GSEA-KEGG enrichment analysis unveiled that the CXCL10 high-expression group was significantly enriched in the cell adhesion molecules pathway and the chemokine signaling pathway (Figure 7A). Conversely, the CXCL10 low-expression group was significantly enriched in the TGF-β and Wnt signaling pathways (Figure 7D). Notably, both the EDNRB high-expression group and the MMP13 low-expression group were significantly enriched in the neuroactive ligand-receptor interaction pathway and the peroxisome proliferator-activated receptor signaling pathway (Figures 7B,F). In contrast, the EDNRB low-expression group and the MMP13 high-expression group were significantly enriched in the base excision repair pathway and leishmaniasis infection pathway (Figures 7C,E). These findings provide key clues for an in-depth elucidation of the specific regulatory networks of CXCL10, EDNRB and MMP13 in the pathogenesis of RA.

### 3.7 Immune cell infiltration and its correlation with core genes

**FIGURE 8:**
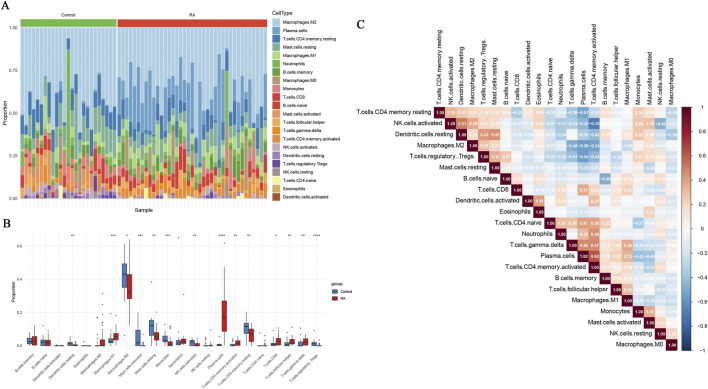
Immune infiltration analysis. **(A)** Stacked bar chart of abundance of immune cells; **(B)** Box plot of differences in immune cell levels between RA and Control groups; **(C)** Correlation heatmap of immune cells. * denotes p < 0.05, ** denotes p < 0.01, *** denotes p < 0.001, **** denotes p < 0.0001.

**FIGURE 9:**
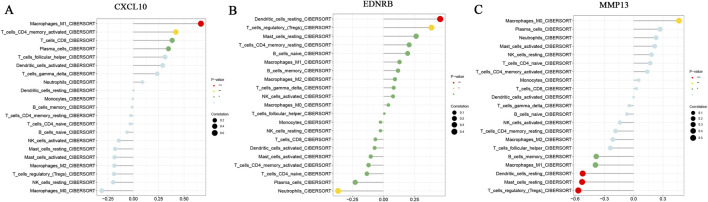
Lollipop plot of correlation analysis between core genes and immune cell infiltration levels. * denotes p < 0.05, ** denotes p < 0.01, *** denotes p < 0.001. **(A)** CXCL10 **(B)** EDNRB **(C)** MMP13.

The CIBERSORT algorithm was leveraged to implement immune infiltration analysis. A stacked histogram was generated to visually display the relative ratios of 22 immune cell subtypes in each sample (Figure 8A). Inter-group comparison unveiled substantial variations in the infiltration patterns of multiple immune cells between the RA and control groups (Figure 8B). In RA synovial tissues, M1 macrophages, plasma cells, activated memory CD4 T cells, and T follicular helper cells were substantially increased. Conversely, M2 macrophages, activated mast cells, monocytes, activated natural killer (NK) cells, and regulatory T cells (Tregs) were markedly decreased. These findings suggested that these immune cell subsets may be engaged in the pathogenesis of RA. Notably, association analysis of immune cells (Figure 8C) indicated a significant positive correlation of activated memory CD4 T cells with plasma cells (r = 0.62), and a significant negative correlation of activated memory CD4 T cells with activated NK cells (r = −0.70). Further analysis revealed that the expressions of the core genes were strongly correlated with the infiltration levels of distinct immune cells. For example CXCL10 was significantly positively correlated with M1 macrophages, activated memory CD4 T cells, and plasma cells (Figure 9A). EDNRB was positively correlated with Tregs (Figure 9B), whereas MMP13 was negatively correlated with both Tregs and M1 macrophages (Figure 9C). These results uncovered that the oxidative stress-related core genes may participate in the pathogenesis of RA by modulating the immune microenvironment.

### 3.8 Drug prediction and molecular docking research for RA treatment

**FIGURE 10:**
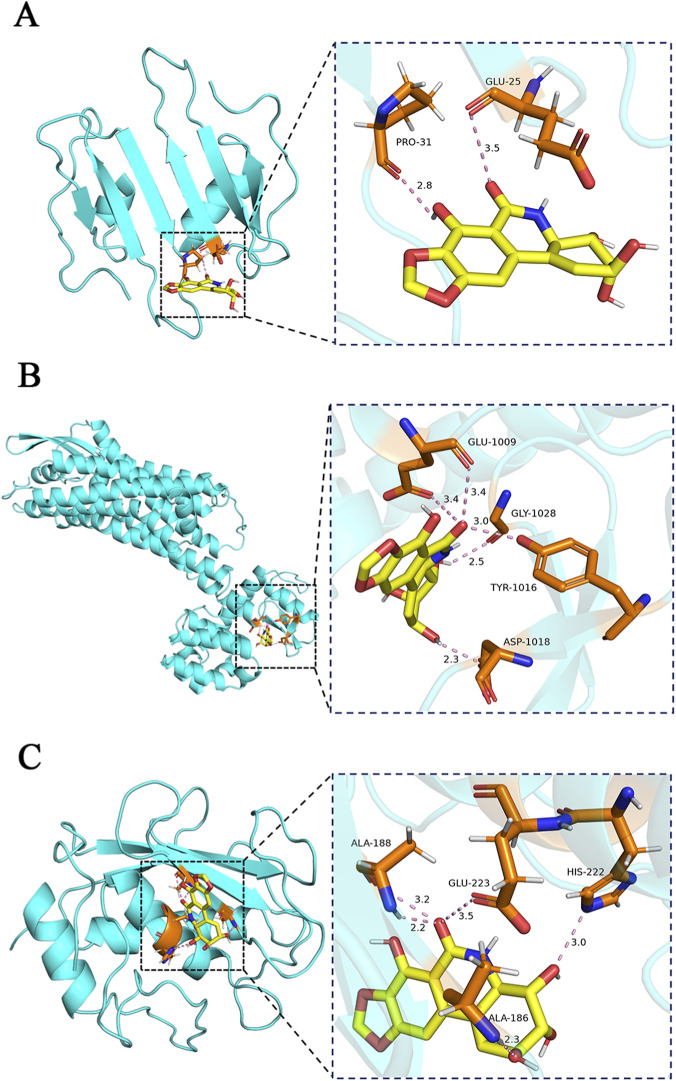
Molecular docking. **(A)** Molecular docking of CXCL10 with narciclasine; **(B)** Molecular docking of EDNRB with narciclasine; **(C)** Molecular docking of MMP13 with narciclasine.

To screen for small-molecule drugs with potential therapeutic effects against RA, we systematically analyzed 137 upregulated and 144 downregulated DEOSGs based on the CMap database. Ten small-molecule compounds with the top negative enrichment scores were ultimately identified, including clofarabine, homoharringtonine, cephaeline, LDN-193189, VU-0418946-1, narciclasine, verrucarin-a, cycloheximide, anisomycin, and tyrphostin-AG-126 (Supplementary Table S3). Among these, narciclasine is an alkaloid extracted from plants of the genus Narcissus within the Amaryllidaceae family (Ceriotti, 1967). It has been confirmed to possess substantial *in vivo* antioxidant and anti-inflammatory activities, demonstrating potent regulatory effects, particularly in inflammatory diseases like arthritis (Nair and van Staden, 2024). Mikami et al. reported that narciclasine significantly alleviated paw swelling in a rat model of adjuvant-induced arthritis (Mikami et al., 1999). These findings suggest that this compound may represent a potential research direction for the development of therapeutic drugs for RA. To further evaluate the affinity of narciclasine for RA-related target proteins, we executed molecular docking experiments. The findings uncovered that the docking scores of narciclasine for CXCL10, EDNRB, and MMP13 were −6.7 kcal/mol, −7.8 kcal/mol, and −7.9 kcal/mol, respectively (Figure 10). The binding energies were all lower than −5.0 kcal/mol. This finding uncovered good affinity with the aforementioned target proteins and the strongest binding observed for MMP13.

### 3.9 Molecular dynamics simulation

**FIGURE 11:**
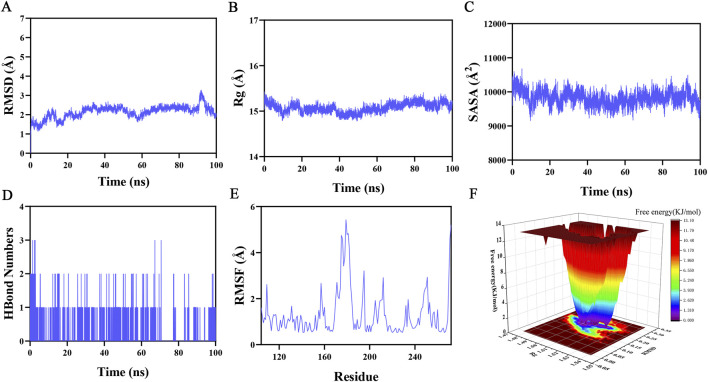
Molecular dynamics simulation. **(A)** RMSD values of the MMP13-narciclasine complex over time; **(B)** Rg values of the MMP13-narciclasine complex over time; **(C)** SASA values of the MMP13-narciclasine complex over time; **(D)** Number of HBonds in the MMP13-narciclasine complex over time; **(E)** RMSF values of the MMP13-narciclasine complex; **(F)** Landscape of free energy.

MMP13, the core target protein with the lowest binding energy, and the small-molecule compound narciclasine were selected to perform a 100-ns molecular dynamics simulation to further validate the molecular docking results and characterize the molecular dynamic behavior. RMSD, as a classic index for examining the conformational stability of proteins and ligands, served to evaluate the displacement of atomic positions from their original coordinates. A smaller displacement indicated higher conformational stability. The system equilibration assessment based on RMSD showed that the complex fluctuated stably from 60 to 90 ns (Figure 11A). A slight increasing trend was observed in the late stages of the simulation, but the fluctuation amplitude remained below 3.2 Å. Rg analysis revealed that the complex showed relatively stable fluctuations in the simulation (Figure 11B). This result suggested no substantial expansion or contraction throughout the dynamics process. The SASA of the complex system fluctuated around 10,000 Å^2^ without substantial changes (Figure 11C), which suggested that ligand binding had a minimal effect on the protein’s spatial structure. HBonds play a crucial role in ligand-protein binding. During the dynamics process, the number of HBonds between narciclasine and the target protein ranged from 0 to 3, and the complex formed approximately one HBond in most instances (Figure 11D). This suggested good HBond interactions between the ligand and the target protein. The RMSF values of the complex were relatively small, with the majority less than 5 Å (Figure 11E), suggesting low structural flexibility and high structural stability. A 3D free energy landscape (FEL) was generated to further characterize the dynamic behavior of the complex (Figure 11F).

### 3.10 Expression of core genes in an *in vitro* cell model

**FIGURE 12:**
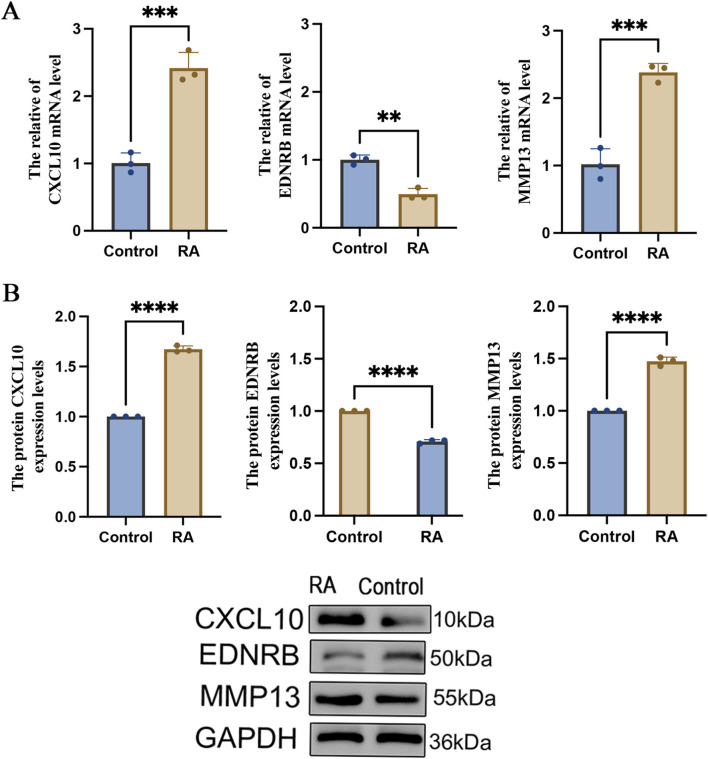
Validation of core gene expression in the *in vitro* cell model. **(A)** qRT-PCR for detecting the mRNA expression levels of CXCL10, EDNRB and MMP13 in the cell model (n = 3); **(B)** Western blot (WB) for detecting the protein expression levels of CXCL10, EDNRB and MMP13 in the cell model (n = 3). ** denotes p < 0.01, *** denotes p < 0.005, **** denotes p < 0.001.

The qRT-PCR and Western blot experiments were executed utilizing human rheumatoid arthritis synovial fibroblast cells to corroborate the expression patterns of the core genes *in vitro*. The findings uncovered that in comparison to the control group, mRNA expression levels of CXCL10 and MMP13 were substantially enhanced in the rheumatoid arthritis synovial fibroblast cells, whereas the mRNA expression of EDNRB was markedly reduced (Figure 12A). The protein-level detection results were in strong agreement with the transcriptional findings. The protein expressions of both CXCL10 and MMP13 were considerably greater than those of the control group, while the expression of EDNRB was markedly downregulated (Figure 12B). Complete WB band information is provided in Supplementary Figure S1. These experimental results were highly consistent with the bioinformatic predictions, thus confirming the differential expression patterns of the three core genes.

## 4 Discussion

RA is a clinically common chronic autoimmune condition (Zhang et al., 2023). Recently, the prevalence of this disease has shown an increasing trend annually, posing a great burden on global healthcare systems (Collaborators, 2023). Concurrently, substantial challenges remain in the early accurate diagnosis and effective intervention of RA. Therefore, it is imperative to explore new molecular pathways to aid in its diagnosis and treatment. Oxidative stress refers to a pathological state featuring an imbalance between the production and clearance of ROS, leading to oxidative damage in cells and tissues (Kondo et al., 2023; Nabih et al., 2024). Oxidative stress exerts an essential effect in the onset and progress of RA and the exploration of potential therapeutic strategies. The current research utilized bioinformatics approaches to screen for OSGs that could serve as diagnostic biomarkers and therapeutic targets. It intended to offer novel perspectives on the understanding of the pathogenesis of RA and the expansion of therapeutic directions.

In the current research, GO and KEGG functional enrichment analysis were implemented on the 281 DEOSGs. The findings unveiled that these genes were markedly clustered in classic immune-inflammatory core pathways of RA, particularly in the MAPK, AMPK, TNF, and Toll-like receptor signaling pathways. Previous research has confirmed that the TNF signaling pathway, as the core initiator of inflammation in RA, can induce the massive secretion of proinflammatory cytokines and facilitate the abnormal proliferation of synovial fibroblast cells by activating the NF-κB and MAPK pathways (Nagar et al., 2010; Webster and Vucic, 2020; Farrugia and Baron, 2016). TNF antagonists have been proven to markedly delay the progression of RA (Xiao et al., 2025). The p38 and JNK subtypes of the MAPK pathway can be directly activated by ROS, thereby regulating the expressions of proinflammatory factors and MMPs (Gaestel, 2015; Whitaker and Cook, 2021). This mechanism, in turn, accelerates the destruction of cartilage and bone tissues (Kanai et al., 2020). The AMPK pathway, serving as a regulatory hub for energy metabolism and inflammatory balance, can alleviate excessive oxidative stress and inflammatory responses in RA when activated, via the inhibition of ROS production and NF-κB pathway activation (Chen et al., 2020; Chen et al., 2018; Jing et al., 2023). The enrichment of DEOSGs in this pathway suggests their potential involvement in this protective mechanism. Furthermore, the Toll-like receptor pathway, as a core regulatory pathway of innate immunity (Elshabrawy et al., 2017; Kawai and Akira, 2010), when activated by damage-associated molecular patterns (DAMPs) in the RA joint microenvironment (Duffy and O'Reilly, 2016), can result in a synergistic effect with the TNF and MAPK pathways through the MyD88-dependent pathway (Huang and Pope, 2009), thus exacerbating chronic inflammatory infiltration in synovial tissue. The co-enrichment of DEOSGs in these core pathways reveals that OSGs can deeply participate in the immune dysregulation and tissue damage processes of RA through the synergistic regulation of multiple pathways.

Subsequently, three machine learning algorithms were adopted to determine three OSGs: CXCL10, EDNRB, and MMP13. A nomogram model based on these genes revealed good diagnostic robustness in both the training set and an external validation set. CXCL10 is a critical inflammatory chemokine and exerts a substantial proinflammatory effect in the onset and progression of RA (Rokni et al., 2025). It primarily functions by specifically binding to its receptor, CXCR3, to recruit immune cells such as T helper 1 (Th1) cells and NK cells to infiltrate the synovial tissue of the joint, thereby amplifying the local inflammatory response (Tokunaga et al., 2018; Lee et al., 2009; Ran et al., 2022). Concurrently, CXCL10 can promote osteoclastogenesis by regulating the expression of RANKL, which in turn accelerates cartilage and bone destruction in the joints of RA patients (Lee et al., 2017; Kang et al., 2021). Research by Pandya et al. has also verified that the expression level of CXCL10 is closely correlated with clinical disease activity of RA (Pandya et al., 2017). CXCL10 has the potential to act as a biomarker for assessing disease activity and a promising therapeutic target in RA (Yoshida et al., 2012). EDNRB is a G protein-coupled receptor and exerts its biological effects mainly by binding to ligands of the endothelin family (Wang et al., 2024; Lange et al., 2007). The binding of endothelin-1 to EDNRB can activate downstream signaling pathways encompassing NF-κB and MAPK, thereby regulating the secretion of proinflammatory cytokines (Bouallegue et al., 2007; Rokni et al., 2022). Furthermore, the altered EDNRB expression in RA may be directly involved in the transmission and perception of nociceptive signals (Cortes-Altamirano et al., 2020). MMP13 is a crucial member of the MMP family (Boldeanu et al., 2023). Proinflammatory factors generated by synovial inflammation in RA can substantially upregulate the MMP13 expression (Goldring et al., 2011; Sylvester et al., 2004). The accumulation of ROS can also activate MMP13 (Del Carlo et al., 2007). Excessive MMP13 accelerates the cleavage of type II collagen and the degradation of proteoglycans, thereby impairing the structural integrity of articular cartilage and accelerating its degeneration (Mehana et al., 2019; Monfort et al., 2006). The resulting cartilage fragments, upon phagocytosis by type A synovial fibroblasts, induce the secretion of various proinflammatory cytokines, thus forming a vicious cycle of inflammation and cartilage degradation that perpetuates RA pathogenesis (Hu and Ecker, 2021).

The regulatory effect of the immune microenvironment in the pathogenesis of RA is attracting considerable research interest. Our immune infiltration analysis uncovered substantial variations in the infiltration patterns of macrophages, T cells, and plasma cells between RA and healthy synovial tissues. Specifically, the ratio of proinflammatory macrophages (M1) was substantially elevated in RA, while the ratio of anti-inflammatory macrophages (M2) was markedly decreased, exhibiting a polarization towards a proinflammatory phenotype (Cutolo et al., 2022; Tian et al., 2025). These polarized macrophages continuously release inflammatory factors, driving the abnormal proliferation and invasion of synovial fibroblasts (Zheng et al., 2024). Following gene rearrangement, T cell receptor recognition, and clonal selection, T cells develop into CD4^+^ T cells and CD8^+^ T cells (Krangel, 2009; Smolen et al., 2016; Riitano et al., 2023). Among them, activated CD4^+^ T cells can further develop into various effector T cell subsets. Th17 cells are a core effector subset mediating pathological damage in RA, exacerbating inflammatory responses through the secretion of characteristic cytokines (Yamada, 2023; Yamada, 2010). Tregs, as a crucial subset of CD4^+^ T cells, possess central immunosuppressive functions (Wang et al., 2023). During the pathological process of RA, the generation and function of Tregs are markedly suppressed, rendering them unable to effectively regulate the overactivated autoimmune response (Yan et al., 2022). Such immune dysregulation further exacerbates the imbalance of local immune homeostasis in the synovial microenvironment. Studies indicate that Treg-based therapy holds promise for effectively alleviating arthritic pathological damage (Yan et al., 2022). Furthermore, highly infiltrating plasma cells may cause the continuous production of autoantibodies like anti-cyclic citrullinated peptide antibodies, promoting the formation and deposition of immune complexes, activating the complement system, and thereby exacerbating damage to joint tissues (Wu et al., 2020; Song et al., 2024). Therefore, the precise regulation of immune cells represents a potentially important strategy in RA treatment.

Finally, we identified narciclasine via the CMap database. As a natural alkaloid compound, narciclasine has been confirmed to possess various biological properties like anti-inflammatory, anti-proliferative, and anti-tumor functions (Sung et al., 2025; Jiang et al., 2025). Preliminary investigations indicate that it functions by modulating signaling pathways including AMPK and NF-κB (Nair and van Staden, 2024; Tian et al., 2022), which are highly aligned with the core regulatory pathways involved in the pathogenesis of RA. Molecular docking and dynamics simulations were performed for narciclasine and the identified RA target proteins, revealing good binding affinity. The formed complex showed an overall certain degree of stability. However, a slight fluctuation in RMSD was observed in the later stages of the dynamics simulation. This fluctuation may introduce some interference to the stability of the results. Based on this, in subsequent drug development, the binding affinity between narciclasine and the target proteins could be enhanced through structural modification of the small-molecule drug (Konstantinidou et al., 2023). Alternatively, optimizing the binding interface at the protein level by considering the 3D structural features of the target proteins could further improve the kinetic stability of the small molecule-target protein complex (Marchand et al., 2025). Furthermore, narciclasine exhibits concentration-dependent cytotoxicity (Sung et al., 2025). Future research could employ strategies like precise concentration control, formulation modifications like nano-drug carriers, localized joint or target cell-specific delivery, and low-dose combination with classical anti-rheumatic drugs (Xiang et al., 2025; Pourmadadi et al., 2025; Nasra et al., 2022). These approaches aim to reduce systemic exposure and off-target effects, thereby mitigating its cytotoxicity. In conclusion, the predictive results of the current research provide important clues for further exploration of the targeted therapeutic value of narciclasine in RA. They also provide a theoretical basis and potential research directions for the development of novel therapeutic drugs for RA.

The current research has several limitations. First, all data used were derived from public databases, and the sample size was relatively limited. Future validations with larger-scale, multi-center cohorts are required to ensure the general applicability of the findings. Second, the research merely corroborated the expression changes of the core genes in a primary synovial fibroblast model utilizing qRT-PCR and Western blot experiments, and no functional experiments were executed. Future studies will be directed toward the systematic experimental validation of these core genes in a variety of cellular and animal models, as well as in-depth exploration of the molecular mechanisms by which they regulate oxidative stress in RA. Finally, foundational experimental evidence for the effect of narciclasine against RA remains scarce at present. Future *in vitro* and *in vivo* basic experiments specifically for RA should be executed. Its biological effects and molecular targets should be systematically investigated, thereby providing a more robust theoretical and experimental basis for the subsequent development and clinical translation of this candidate drug. In summary, the conclusions drawn in the current research remain exploratory in terms of the general applicability and potential clinical translational value until more confirmatory experimental evidence and clinical data are obtained. Further in-depth research should be undertaken to validate, refine, and expand these findings.

## 5 Conclusion

By integrating bioinformatics analysis with multiple machine learning algorithms, the current research identified three OSGs, namely CXCL10, EDNRB, and MMP13. The three genes have the potential to serve as biomarkers for RA. Furthermore, the small-molecule drug narciclasine was screened through molecular docking and molecular dynamics simulations, which exhibits certain research value for RA treatment. The results offer novel perspectives on the understanding of the pathogenesis of RA. They also offer valuable references for the molecular diagnosis, targeted intervention, and the development of candidate drugs for RA.

## Data Availability

The datasets presented in this study can be found in online repositories. The names of the repository/repositories and accession number(s) can be found in the article/Supplementary Material.

## Supplementary material

SUPPLEMENTARY FIGURE 1 Western blot images from three independent biological replicates.
