# Supplementary figures and images for: Screening of potential oxidative stress-related biomarkers and therapeutic drugs in rheumatoid arthritis based on integrative bioinformatics, machine learning, and molecular dynamics simulations
Source: Front Mol Biosci. 2026 Mar 23;13:1804435. doi: 10.3389/fmolb.2026.1804435 (PMC13050699; doi:10.3389/fmolb.2026.1804435)

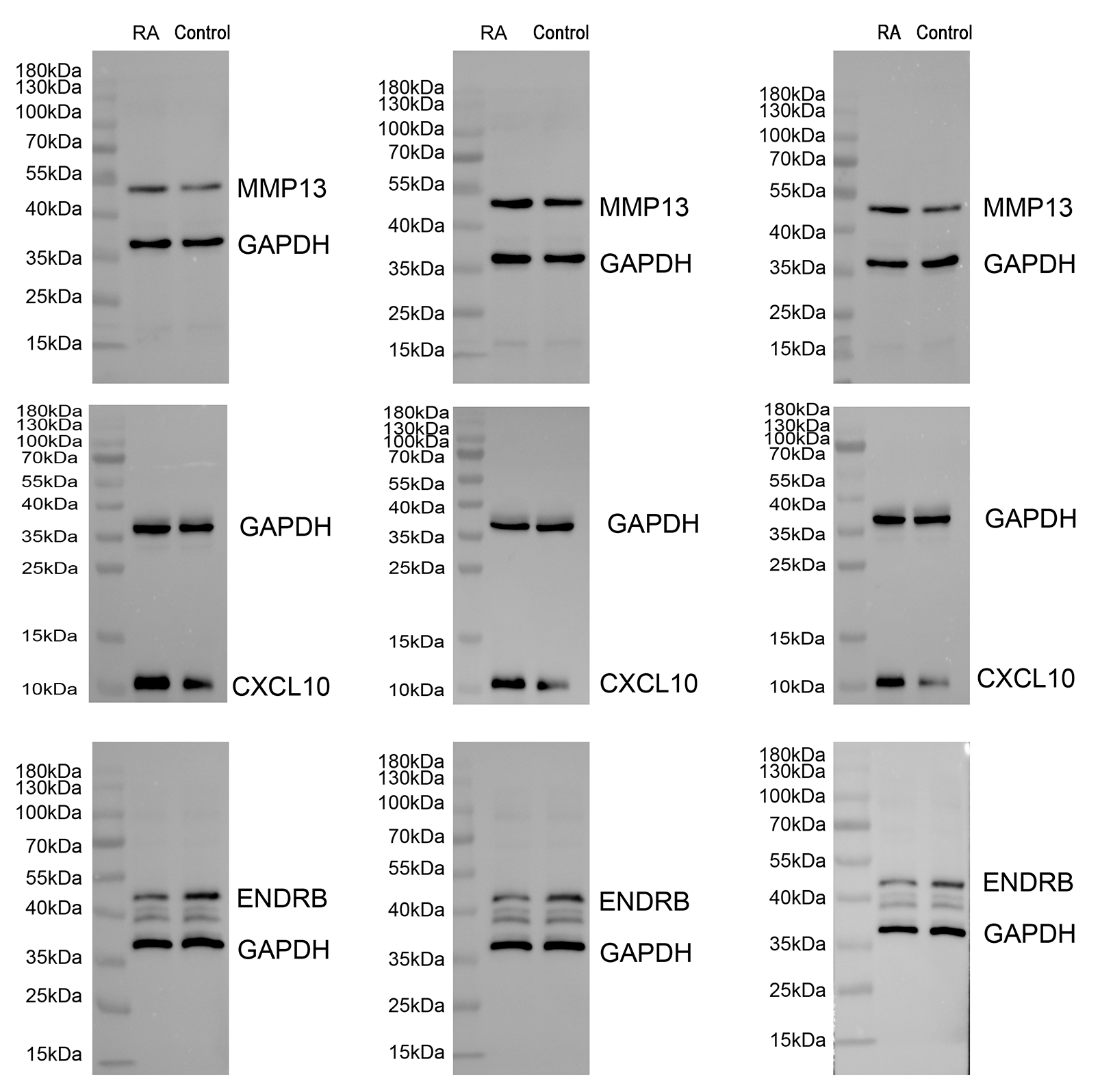

Supplement: Supplementary file 3 [file Image1.tif]
